# TGFβ1-induced hedgehog signaling suppresses the immune response of brain microvascular endothelial cells elicited by meningitic *Escherichia coli*

**DOI:** 10.1186/s12964-023-01383-y

**Published:** 2024-02-15

**Authors:** Jinrui Sun, Ruicheng Yang, Jiyang Fu, Dong Huo, Xinyi Qu, Chen Tan, Huanchun Chen, Xiangru Wang

**Affiliations:** 1 National Key Laboratory of Agricultural Microbiology, College of Veterinary Medicine, Huazhong Agricultural University, Wuhan, 430070 China; 2 Key Laboratory of Preventive Veterinary Medicine in Hubei Province, The Cooperative Innovation Center for Sustainable Pig Production, Wuhan, 430070 China; 3 Frontiers Science Center for Animal Breeding and Sustainable Production, Wuhan, 430070 China; 4 International Research Center for Animal Disease, Ministry of Science and Technology of the People′s Republic of China, Wuhan, 430070 China

**Keywords:** TGFβ1, Hedgehog signaling, SAG, Brain microvascular endothelial cells, miR-155, Neuroinflammation, *Escherichia coli*

## Abstract

**Background:**

Meningitic *Escherichia coli* (*E. coli*) is the major etiological agent of bacterial meningitis, a life-threatening infectious disease with severe neurological sequelae and high mortality. The major cause of central nervous system (CNS) damage and sequelae is the bacterial-induced inflammatory storm, where the immune response of the blood-brain barrier (BBB) is crucial.

**Methods:**

Western blot, real-time PCR, enzyme-linked immunosorbent assay, immunofluorescence, and dual-luciferase reporter assay were used to investigate the suppressor role of transforming growth factor beta 1 (TGFβ1) in the immune response of brain microvascular endothelial cells elicited by meningitic *E. coli*.

**Result:**

In this work, we showed that exogenous TGFβ1 and induced noncanonical Hedgehog (HH) signaling suppressed the endothelial immune response to meningitic *E. coli* infection via upregulation of intracellular miR-155. Consequently, the increased miR-155 suppressed ERK1/2 activation by negatively regulating KRAS, thereby decreasing IL-6, MIP-2, and E-selectin expression. In addition, the exogenous HH signaling agonist SAG demonstrated promising protection against meningitic *E. coli*-induced neuroinflammation.

**Conclusion:**

Our work revealed the effect of TGFβ1 antagonism on *E. coli*-induced BBB immune response and suggested that activation of HH signaling may be a potential protective strategy for future bacterial meningitis therapy.

Video Abstract

**Supplementary Information:**

The online version contains supplementary material available at 10.1186/s12964-023-01383-y.

## Background

Bacterial meningitis is an important life-threatening infection disease, especially affecting young children and the elder with low immunity [1]. *Escherichia coli* (*E. coli*) is the most common gram-negative bacillary organism causing meningitis [2]. Most cases of bacterial meningitis are initiated through hematogenous spread, where circulating pathogenic bacteria penetrate the blood-brain barrier (BBB) [3]. Consequently, patients suffer from central nervous system (CNS) disorder attributed to BBB and pathogen-induced neuroinflammation [4, 5].

The BBB is an essential physiologic barrier that separates CNS from soluble inflammatory mediators and immune cells in peripheral circulation [6]. Brain microvascular endothelial cells (BMECs) are critical regulators of the barrier’s immune reactivity and CNS immune quiescence, making them a core component of the BBB [7, 8]. Physiologically, the BMECs control and modulate the impact of peripheral immune events on the CNS to protect the brain [9]. Upon stimulation of DAMP and PAMP, the BMECs actively participate in various CNS immune events by upregulating selectins, cell adhesion molecules (CAMs), and inflammatory mediators, such as interleukins, C-C motif ligands, and C-X-C motif ligands [10]. For instance, in experimental autoimmune encephalomyelitis, the MIP-2 secreted by activated BMECs was crucial for early inflammation initiation and leukocyte breaching the BBB [11, 12]. Furthermore, BMECs-secreted IL-6 promotes inflammatory astrocytes expressing sCp, enhances Fe^2+^ transfer into CNS, subsequently leading to oxidative stress and neural degeneration in the brain [13, 14]. Additionally, E-selectin, a well-known pro-inflammatory mediator derived from BMECs, has a protective effect on cerebral ischemia in stroke studies. E-selectin knockout MCAO mice exhibited improved neurological function, reduced infarct area, and decreased the number of apoptosis cells and IL-1β content [15]. Furthermore, mucosal tolerance to E-selectin in MCAO mice provided cell-mediated protection against ischemic brain injury [16].

TGFβ1 and its downstream transcription factors, Smads participate in various physiological and pathological processes, particularly in immune reaction modulation. In dermatitis research, it was observed that TGFβ1 overexpression in mouse epidermis induced inflammatory skin lesions and Koebner’s phenomenon, indicating the pro-inflammatory role of TGFβ1 [17]. Inversely, TGFβ1 treatment reportedly reduced susceptibility to experimental autoimmune encephalomyelitis and suppressed cytokines production. TGFβ1 knockout mice died from multifocal inflammation and autoimmune disorders in internal organs, suggesting its immunosuppressive effect in these organs [18]. Besides modulating the target genes directly with Smads proteins, TGFβ1 also engages in crosstalk with other signaling axes, such as PAR1, PAR2, miR-145, let-7d, and miR-18, and impacting multiple biological processes indirectly [19, 20]. Previously, we presented the crosstalk between TGFβ1 signaling and hedgehog (HH) signaling in BMECs. Astrocytes-derived TGFβ1, and the exogenous recombined TGFβ1 (rTGFβ1) activate transcription factors of HH signaling, Gli1 and Gli2. This noncanonical HH signaling protects mice from death upon meningitic *E. coli* infection by maintaining BBB integrity [21]. Consideration of TGFβ1 to modulate the immunity, we presumed that TGFβ1 might also repress the BBB immune reaction while maintaining the BBB integrity.

This study demonstrated that the TGFβ1-HH cascade repressed BMECs’ immune reaction upon *E. coli* infection. In vivo, exogenous rTGFβ1 treatment protected mice from meningitic *E. coli* challenge and reduced IL-6, MIP-2, and E-selectin expression in the brain. In vitro study revealed that TGFβ1-HH promoted miR-155 expression in BMECs, which negatively regulated KRAS and repressed ERK1/2 phosphorylation induced by *E. coli*. We revealed that activating TGFβ1-HH signaling by rTGFβ1 or SAG in BMECs suppressed the bacteria-induced immune reaction and protected CNS from meningitic *E. coli*. Understanding the regulatory mechanism of TGFβ1-HH cascades in BMECs can contribute to developing new treatment strategies to reduce CNS damage in bacterial meningitis and other CNS infection-related diseases.

## Methods

### Bacterial strain and cell culture

*E. coli* strain RS218 (O18:K1:H7) was originally obtained from the cerebrospinal fluid of a neonate with meningitis and gifted by Prof. Kwang Sik Kim at Johns Hopkins University School of Medicine. The *E. coli* strain was grown aerobically at 37 °C in Luria–Bertani medium overnight.

The human BMECs (hBMECs) were kindly gifted from Prof. Kwang Sik Kim at Johns Hopkins University School of Medicine, and the Gli-KO hBMECs cell lines were constructed and tested in a previous study [22]. The cells were routinely cultured in RPMI 1640 supplemented with 10% fetal bovine serum (FBS), 2 mM L-glutamine, 1 mM sodium pyruvate, essential amino acids, nonessential amino acids, vitamins, and penicillin and streptomycin (100 U/mL). The HEK-293 T cells (ATCC® CRL-3216™) were cultured in Dulbecco’s Modified Eagle’s Medium with 10% FBS and penicillin and streptomycin (100 U/mL). All cells were cultured in a 37 °C incubator under 5% CO_2_ until reaching monolayer confluence. In some experiments, confluent hBMECs were starved in serum-free medium (1:1 mixture of Ham’s F-12 and 199 medium) for 12–16 h before further treatment.

### Reagents and antibodies

The HH Gli1/2 inhibitor GANT61 and the HH pathway agonist SAG were purchased from MedchemExpress (Monmouth, NJ, USA). The immunofluorescence (IF) staining kits containing Cy3-labeled goat anti-rabbit IgG, FITC-labeled goat anti-rabbit IgG, the 4′,6-diamidino-2-phenylindole (DAPI) reagent, and Fluo-3-AM probe were obtained from Beyotime (Shanghai, China). The anti-KRAS antibody was from Proteintech (Chicago, IL, USA). Anti-ERK1/2, anti-phospho-ERK1/2, anti-JNK, anti-phospho-JNK, anti-p38, anti-phospho-p38, anti-p65, anti-phospho-p65, HRP-conjugated anti-rabbit IgG, and HRP-conjugated anti-mouse IgG antibodies were purchased from Cell Signaling Technology (Danvers, MA, USA). Anti-CD31 antibody for IF was from Abcam (Cambridge, MA, USA). Anti-β-actin and anti-E-selectin antibodies were obtained from HuaAn Biotechnology Co., Ltd. (Hangzhou, China). The lipofectamine 3000 transfection reagent was obtained from Invitrogen (Carlsbad, CA, USA). Human recombinant TGFβ1 and mouse recombinant TGFβ1 were obtained from the R&D system (Minneapolis, MN, USA). Mouse IL-6 ELISA Kits and Mouse MIP-2 ELISA Kits were purchased from Neobioscience (Shenzhen, China). The has-miR-155 mimics, mimics NC, has-miR-155 inhibitors, inhibitors NC, and the primer kit for has-miR-155 qPCR were purchased from JTSBIO CO.,Ltd. (Wuhan, China).

### Mice infection assays

The 21-day-old specific-pathogen-free female Kunming mice were obtained from the Laboratory Animal Services Center, Huazhong Agricultural University (Wuhan, China). For the infection, mice were challenged with *E. coli* strain RS218 via the tail vein at 3 × 10^6^ CFUs [21]. The brains from moribund and control mice were subjected to IF or enzyme-linked immunosorbent assay (ELISA) assays. In some assays, the recombinant TGFβ1 protein or SAG was injected through the tail vein 12 h before the *E. coli* challenge.

### Elisa

Mice were challenged with bacteria and indicated reagents; the brains of five mice from each group with similar symptoms were randomly selected for ELISA determination, using Mouse IL-6 ELISA Kits and Mouse MIP-2 ELISA Kits, following the manufacturers’ instructions.

### Bacterial infection of hBMECs

Following our previously described methods, *E. coli* strain RS218 infection of hBMECs was performed [23]. Briefly, the confluent hBMECs were starved in a serum-free medium for 12–16 h. Overnight *E. coli* cultures were resuspended and diluted in the same serum-free medium and added to the cells at a multiplicity of infection of 10 for the indicated time points. Cells were then washed three times with pre-chilled PBS and collected for RNA isolation using TRIzol reagent or protein extraction with RIPA lysis buffer.

### Western blot

hBMECs cultures were homogenized or lysed in RIPA buffer containing protease inhibitor cocktail, then centrifuged at 15,000×*g* for 60 min at 4 °C to remove the insoluble cell debris. Cell lysates protein concentrations were measured with the BCA protein assay kit, and equivalent protein samples were subjected to Western blot assay as previously described [24].

### Reverse transcription and real-time quantitative polymerase chain reaction (RT-qPCR)

Total RNA was extracted by using the TRIzol reagent, and the RNA purity and concentration were assessed by NanoDrop 2000 Ultramicro spectrophotometer. RT-PCR was performed to generate cDNA using HiScript II Q RT SuperMix for qPCR (+gDNA wiper). The qPCR was performed with qTOWER^3^/G quantitative real-time PCR thermal cycler using MonAmp SYBR Green qPCR Mix following the manufacturer’s instructions. The primers used for RT-qPCR are listed in Table S1. The expression of the target genes was normalized against *GAPDH*, and the has-miR-155 expression was normalized to *U6*. Each assay was performed in triplicate.

### Immunofluorescence

For IF, paraffin sections of the mice’s brains were deparaffinized and rehydrated in xylene and ethanol. IF experiments were performed according to the instructions provided by the relevant kits. Briefly, sections were washed with PBS three times and then fixed with 4% paraformaldehyde for 30 min. The fixed sections were then treated with 1% Triton X-100 in PBS before non-specific site blocking and antibody incubation. E-selectin and KRAS were labeled with Cy3, and CD31 was labeled with FITC. The sections were observed with an ECHO REVOLVE microscope.

### Transfection

HEK-293 T or hBMECs cells grown to 70% confluence were subjected to transfection experiments with Lipofectamine 3000 reagent according to the manufacturer’s instructions. Briefly, 5 μg of plasmids, 10 μL of P3000, 7.5 μL of Lipo3000, and 500 μL of Opti-MEM were mixed gently and incubated at 25 °C for 15 min. The mixture was then added dropwise to the cells in the 6-well plates and incubated at 37 °C with 5% CO_2_ for 24 h.

### Dual-luciferase reporter assay of Gli1/2 binding to *mir155hg* promoter

Before the luciferase reporter assay, the coding sequence (CDS) of human Gli1 and Gli2 were amplified and cloned into the pcDNA3.1(+) vector to generate the overexpression plasmids pcDNA3.1-Gli1 and pcDNA3.1-Gli2. The promoter region of *mir155hg* was amplified and cloned into the firefly luciferase reporter vector pGL3-basic to generate the wild-type reporter plasmids pGL3-mir155hg-promo-WT. Meanwhile, a serial of truncated promoters, and site-directed mutation of promoters, were similarly constructed into pGL3-basic (Fig. 4). All primers used in the dual-luciferase assays are listed in Table S2.

For dual-luciferase reporter assay, the pcDNA3.1 overexpression plasmid, the corresponding pGL3 reporter plasmid, and pRL-TK plasmid were co-transfected into HEK-293 T cells in 24-well plates. Both firefly luciferase activity and renilla luciferase activity were tested after 36 h of transfection by the Dual-Luciferase Reporter assay system with Spark 10 M multimode microplate reader. Relative luciferase activity was calculated by the ratio of reporter activity (firefly fluorescence) to that of control activity (renilla fluorescence), and the results are shown as the representative of three independent assays.

### Dual-luciferase reporter assay of miR-155 binding to kras 3′UTR

The sequences of *kras* 3′UTR and *kras* 3′UTR-mut were synthesized by Genscript and cloned into the psiCHECK-2 plasmid for dual-luciferase activity assay (Fig. 4). Briefly, HEK-293 T cells were seeded in 24-well plates and cultured until 70% confluence before transfection. For each experimental group, 200 ng plasmids and 50 nM of has-miR-155 mimics or negative control were used for the transfection. After 24 h of incubation, cells were collected, and both firefly luciferase activity and renilla luciferase activity were detected using a Dual-Luciferase kit following the manufacturer’s instructions. Results were calculated as the ratio of renilla luciferase activity and the firefly luciferase activity and were recorded as mean ± standard deviation (mean ± SD) from three replicated wells.

### Statistical analysis

Data were expressed as the mean ± standard error of the mean (mean ± SEM) from at least three replicates. The statistical significance of the differences between groups was analyzed by a one-way analysis of variance (ANOVA) or two-way ANOVA embedded in GraphPad Prism, version 6.0 (GraphPad Software Inc., La Jolla, CA, USA). *P* < 0.05 (*) was considered significant, and *p* < 0.01 (**) was considered highly significant.

## Results

### Recombined TGFβ1 repressed meningitic *Escherichia coli* induced blood-brain barrier immune reaction

**Fig. 1:**
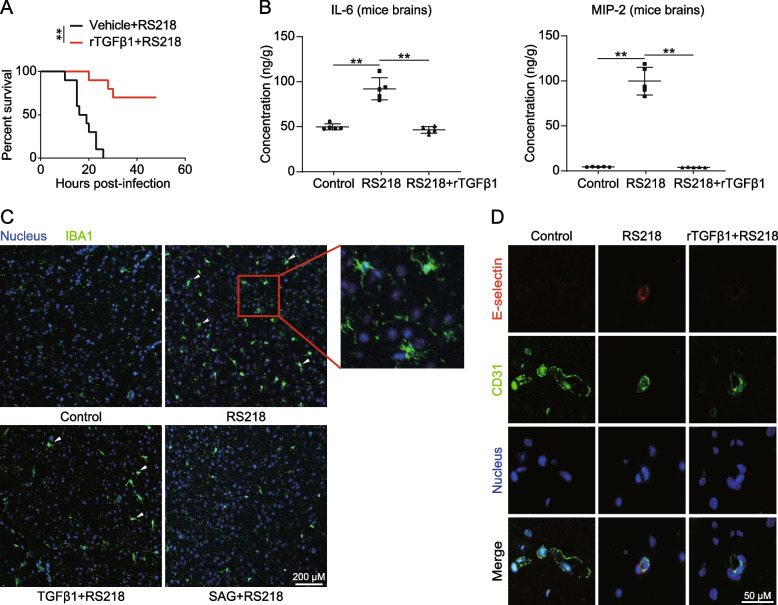
rTGFβ1 protected mice from RS218 and repressed the neuroinflammation. **A** Effects of the rTGFβ1 pre-treatment at 1 μg/kg (for 12 h) on the survival of the mice after the challenge of RS218 (*n* = 10). **p < 0.01. **B** ELISA analysis of IL-6 and MIP-2 in brain lysates from RS218 challenged mice with or without rTGFβ1 pre-treatment at 1 μg/kg (*n* = 5). Data are presented as mean ± SD from five individual mice in each group. **p < 0.01. **C** Mice were challenged with RS218 and pretreated with TGFβ1 (at 1 μg/kg) or SAG (at 10 mg/kg). Microglia were labeled with anti-IBA1 in green, and nuclei were labeled with DAPI in blue. Right panel: high magnifications of the areas delineated by a rectangle. The arrows and the rectangle indicated the typical activated microglia. Scale bar = 200 μm. **D** IF assays demonstrating the E-selectin expression in brains of mice challenged by RS218 with or without rTGFβ1 pre-treatment (at 1 μg/kg). The E-selection was stained in red. CD31 was specifically applied for labeling the micro-vessels in green. The cell nucleus was stained in blue with DAPI. Scale bar = 50 μm

Previously, we observed that rTGFβ1 treatment protected mice from meningitic *E. coli* strain RS218 attack [21]. In this study, we retested the effects of rTGFβ1, and the mice were challenged with RS218 with or without treatment of rTGFβ1 before infection. As presented in Fig. 1A, the mice pretreated with rTGFβ1 were mostly well protected from death. Considering rTGFβ1 immunomodulatory effects, we presumed that rTGFβ1 might protect the mice by repressing the CNS inflammation induced by *E. coli* infection, not just maintaining the BBB integrity as previously presented. To verify this, we tested the levels of the inflammatory mediators IL-6 and MIP-2 in challenged mice brains using ELISA. The mice brain contents of IL-6 and MIP-2 were highly induced by RS218 challenge and ultimately suppressed upon rTGFβ1 treatment (Fig. 1B). Furthermore, microglial activation in RS218-challenged mice brains was inhibited by rTGFβ1 treatment (Fig. 1C). Additionally, we analyzed the expression of E-selectin on the BMECs of mice challenged with RS218 via IF. As shown, E-selectin expression (labeled with Cy3) around the BMECs (labeled with anti-CD31-FITC) was significantly upregulated by infection, while rTGFβ1 treatment prevented the E-selectin induction (Fig. 1D). The data indicated that rTGFβ1 treatment before infection repressed the inflammatory in CNS and protected animals from meningitic *E. coli* infection.

### rTGFβ1-induced HH signaling suppressed ERK1/2 activation in BMECs

**Fig. 2:**
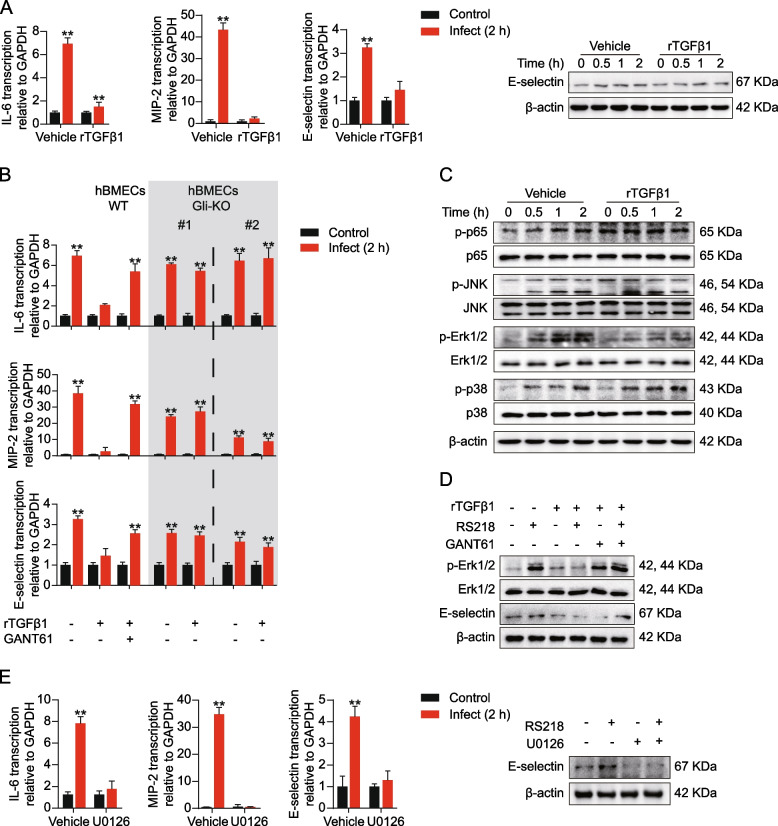
rTGFβ1 inhibited ERK1/2 signaling and immune reaction of RS218 infected hBMECs relying on inducted noncanonical HH signaling. **A** Detecting expression alterations of IL-6, MIP-2, and E-selectin in hBMECs with qPCR or Western blot. The cells were infected with RS218 with or without rTGFβ1 pre-treatment (at 50 ng/mL). **p < 0.01. The qPCR assays were performed in triplicates, and results are presented as mean ± SEM. **B** qPCR detecting IL-6, MIP-2, and E-selectin transcription upon RS218 infection in wild-type hBMECs or Gli-KO hBMECs. The wild-type hBMECs were pretreated with rTGFβ1 (at 50 ng/mL) or together with GANT61 (at 10 μM). The Gli-KO hBMECs were pretreated with rTGFβ1 (at 50 ng/mL). **p < 0.01. The qPCR assays were performed in triplicates, and results are presented as mean ± SEM. **C** Western blot detecting phosphorylation of p65, JNK, ERK1/2, and p38 in RS218 infected hBMECs. The cells were pretreated with or without rTGFβ1 at 50 ng/mL. **D** Western blot detecting expression of E-selectin and ERK1/2 phosphorylation in RS218 infected hBMECs. The cells were pretreated with rTGFβ1 (at 50 ng/mL) or GANT61 (at 10 μM). **E** Detecting expression alterations of IL-6, MIP-2, and E-selectin in hBMECs with qPCR or Western blot. The cells were infected with RS218 with or without U0126 treatment (at 5 μM). **p < 0.01. The qPCR assays were performed in triplicates, and results are presented as mean ± SEM

BBB was the key regulator of CNS immunity events, and it has been demonstrated that rTGFβ1 treatment reduces E-selectin expression in BMECs. The phenotype indicated that rTGFβ1 treatment suppressed the endothelial immune reaction. To confirm this, we tested IL-6, MIP-2, and E-selectin expression in hBMECs challenged with RS218, and the cells were pretreated with or without rTGFβ1. As presented in Fig. 2A, rTGFβ1 treatment repressed the inflammatory mediators’ transcription because of infection, and E-selectin protein expression was also attenuated. Previously, we have demonstrated rTGFβ1 induces noncanonical HH signaling in hBMECs [22]. We further suspected that such immunosuppression effects of rTGFβ1 were HH-dependent. To test this, we employed HH inhibitor GANT61, and the reagent blocked the rTGFβ1 effects on hBMECs, and rTGFβ1 treatment was incapable of reducing the upregulation of IL-6, MIP-2, and E-selectin upon RS218 infection. Furthermore, when HH signaling was disabled with Gli knockout, rTGFβ1 also failed to repress the inflammatory mediators’ expression in hBMECs (Fig. 2B). These results indicated that the TGFβ1 immunosuppression effects on BMECs relied on the inducted noncanonical HH signaling.

**Fig. 3:**
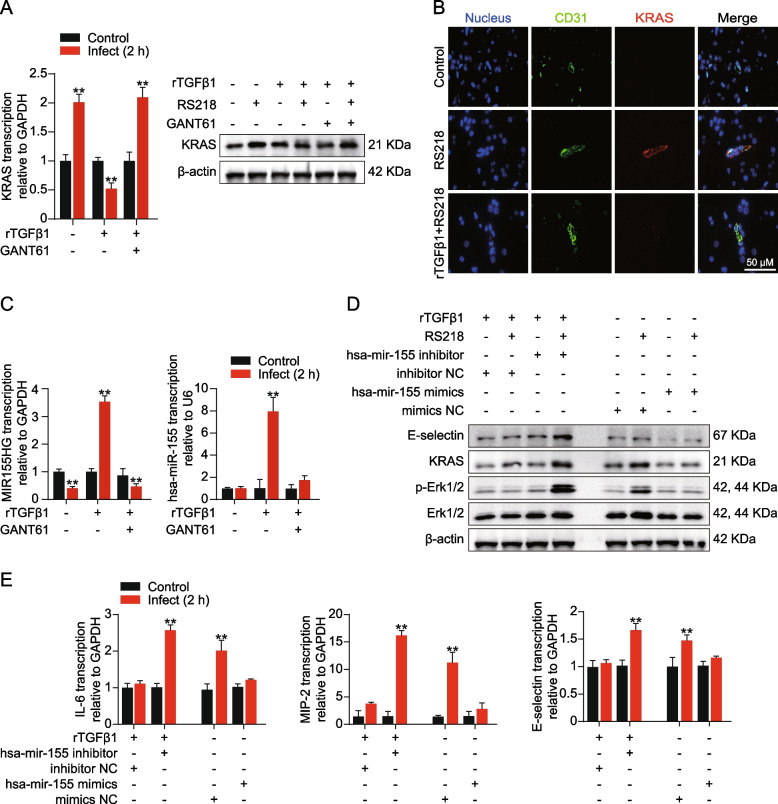
TGF-HH axis suppressed ERK1/2 signaling in RS218 infected hBMECs via modulating miR155 and KRAS. **A** Western blot and qPCR detecting expression of KRAS in RS218 infected hBMECs. The cells were pretreated with rTGFβ1 (at 50 ng/mL) or GANT61 (at 10 μM). **p < 0.01. The qPCR assays were performed in triplicates, and results are presented as mean ± SEM. **B** IF assays showing the KRAS expression in BMECs of mice challenged by RS218 with or without rTGFβ1 pre-treatment (at 1 μg/kg). The KRAS was stained in red. CD31 was specifically applied for labeling the micro-vessels in green. The cell nucleus was stained in blue with DAPI. Scale bar = 50 μm. **C** qPCR detecting MIR155HG and miR-155 expression upon RS218 infection in hBMECs. The cells were pretreated with rTGFβ1 (at 50 ng/mL) or GANT61 (at 10 μM). **p < 0.01. The qPCR assays were performed in triplicates, and results are presented as mean ± SEM. **D** Western blot detecting expression of E-selectin, KRAS and ERK1/2 phosphorylation in RS218 infected hBMECs with or without rTGFβ1 pre-treatment (at 50 ng/mL). The cells were transfected with has-miR-155 mimics, mimics NC, has-miR-155 inhibitors, or inhibitors NC at 50 nM as indicated. **E** qPCR detecting expression alterations of IL-6, MIP-2, and E-selectin in RS218 infected hBMECs with or without rTGFβ1 pre-treatment (at 50 ng/mL). The cells were transfected with has-miR-155 mimics, mimics NC, has-miR-155 inhibitors, or inhibitors NC at 50 nM as indicated. **p < 0.01. The qPCR assays were performed in triplicates, and results are presented as mean ± SEM

Further, we intended to explore the molecular mechanism of immunosuppression by the TGF-HH axis. We tested the NFκB signaling, P38 signaling, JNK signaling, and ERK1/2 signaling activity in hBMECs upon infection, as these pathways are recognized as classical immunity-related signaling. As presented in Fig. 2C, ERK1/2 phosphorylation in hBMECs responding to RS218 infection was impeded with rTGFβ1 presence. This impedance was reversed by GANT61, which also suggested the core role of noncanonical Hh signaling in the immunosuppression of rTGFβ1 (Fig. 2D). Additionally, once ERK1/2 phosphorylation was blocked with U0126, we also observed that IL-6, MIP-2, and E-selectin upregulation was attenuated (Fig. 2E). Taking together, the data proved the concept that rTGFβ1 represses the hBMECs immune reaction induced by meningitic *E. coli* via by blocking the ERK1/2 phosphorylation, and this process mainly relies on the noncanonical HH signaling inducted by rTGFβ1.

### TGF-HH axis reduces ERK1/2 activation by upregulating miR-155

**Fig. 4:**
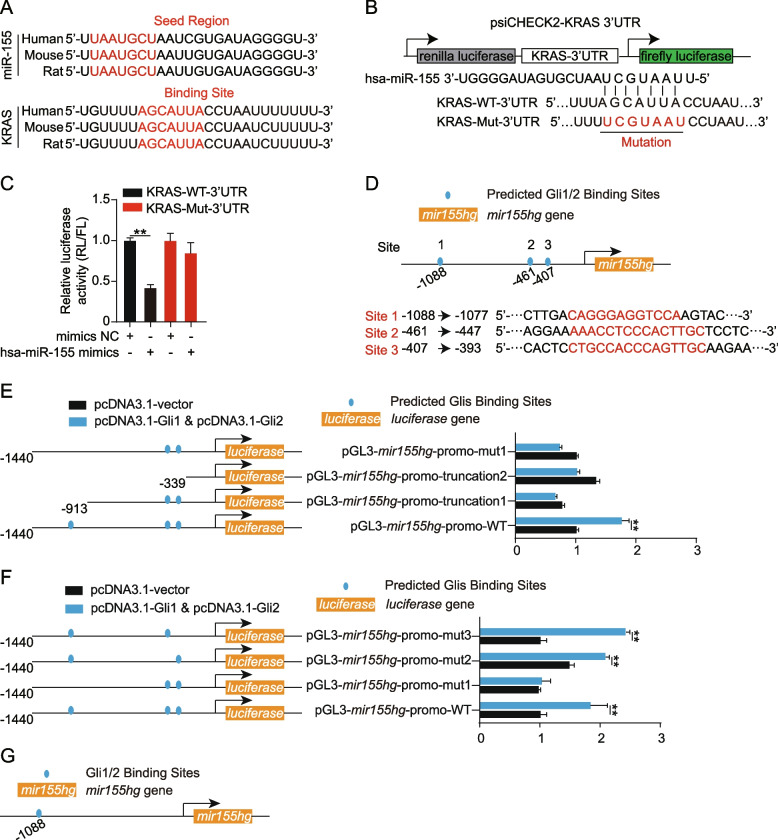
Analysis of miR-155 binding sites on *kras* 3’UTR and Gli1/2 binding sites on mir155hg promotor by dual-luciferase reporter assays. **A** Conservation of the miR-155 sequence among different species (upper panel), and conservation of the miR-155 target sequence in KRAS among different species (lower panel). Human, *Homo sapiens*; Mouse, *Mus musculus*; Rat, *Rattus norvegicus*. **B** The miRNA response elements (MREs) of miR-155 were shown on the sequence of kras 3′-UTR, and mutations were introduced on these MREs. Both wild-type and mutated sequences were cloned into the psiCHECK-2 plasmid. **C** dual-luciferase reporter assay testing miR-155 binding with kras 3’UTR. HEK-293 T cells were co-transfected with has-miR-155 mimics or mimics NC as control oligonucleotide (final concentration at 50 nM) together with the wild-type (KRAS-WT-3’UTR) or mutated (KRAS-Mut-3’UTR) kras 3′-UTR luciferase reporter plasmids. The renilla luciferase activity was measured and normalized to firefly luciferase activity after 36 h. **p < 0.01. The assays were performed in triplicates, and results are presented as mean ± SEM. **D** Schematic of the three predicted Gli1/2 binding sites on mir155 hg promotor (upper panel) and their binding sequences accordingly (lower panel). The binding sites were located at − 1088 to − 1077 (site 1), − 461 to − 447 (site 2), and − 407 to − 393 (site 3) of the mir155hg promotor. The gli2 luciferase activities were tested by applying a series of truncations (**E**) as well as site-targeted mutations (**F**) on the mir155hg promoter, along with pcDNA3.1-Gli1 and pcDNA3.1-Gli2 and pRL-TK plasmids. The specific constructs used in the truncation assays (**E**) included pGL3-basic vector, pGL3-mir155hg-promo-WT (containing promotor region from − 1440 to + 226), pGL3-mir155hg-promo-truncation1 (from − 913 to + 226) and pGL3-mir155hg-promo-truncation2 (from − 339 to + 226). The specific constructs used in the site-mutation assays (**F**) included pGL3-basic vector, pGL3- mir155hg-promo-WT (containing all 3 sites), pGL3-mir155hg-promo-mut1 (lack of site 1), pGL3-mir155hg-promo-mut2 (lack of site 2) and pGL3-mir155hg-promo-mut3 (lack of site 3). The luciferase activities were determined and presented as the ratio of firefly and renilla luciferase activity. The assays were performed with three replicates and data are presented as mean ± SEM. **p < 0.01. **G** Schematic of the Gli1 and Gli2 binding to the mir155hg promotor at around − 1088 in hBMECs

KRAS is a well-known initiator of ERK1/2 activation in Ras/Raf/MEK/ERK cascade [25]. We wondered if KRAS was involved in the ERK1/2 repression by rTGFβ1. As shown in Fig. 3A, KRAS in hBMECs was downregulated upon infection because of rTGFβ1 treatment, while it was upregulated in the presence of GANT61. Consistently in vivo, KRAS (labeled with Cy3) in BMECs (labeled with anti-CD31-FITC) of mice treated with TGFβ1 prior infection was downregulated, in contrast to those left untreated (Fig. 3B). The data suggested that KRAS participated in the ERK1/2 repressing because of rTGFβ1. According to sequence analysis, KRAS was the potential target gene of miR-155 [26]. We detected the expression of miR-155 and its precursor MIR155HG. It turned out that miR-155 was upregulated in hBMECs upon infection with rTGFβ1 treatment, while it was decreased in hBMECs treated with GANT61 or untreated. Consistently, transcription of MIR155HG was also increased in the presence of rTGFβ1 and decreased when cells were treated with GANT61 (Fig. 3C). Therefore, we presumed that rTGFβ1 might decrease KRAS by promoting miR-155 expression. To verify, we transfected the hBMECs with hsa-miR-155 inhibitors and hsa-miR-155 mimics. As shown in Fig. 3D, hsa-miR-155 inhibitors blocked the rTGFβ1 immunosuppression in hBMECs, resulting in the upregulation of KRAS and phosphorylation of ERK1/2 in hBMECs. Additionally, the downstream decrease in IL-6, MIP-2, and E-selectin were also reversed (Fig. 3D-E). Contrarily, has-miR-155 mimics repressed the KRAS expression and ERK1/2 activity in hBMECs upon infection, subsequently attenuating the expression of IL-6, MIP-2, and E-selectin (Fig. 3D-E). Furthermore, sequence alignment demonstrated that the seed region of miR-155 (5′-UAAUGCU-3′) might bind to *kras* gene 3’UTR region at 5′-AGCAUUA-3′, which was conserved among different species, including human, mouse, and rat (Fig. 4A). To test the possible regulation between miR-155 and *kras* gene 3’UTR region, the dual-luciferase reporter plasmid was constructed by cloning *kras* gene 3’UTR binding region into the psiCHECK-2 plasmid, name after KRAS-WT-3’UTR. Likewise, a mutant 3’UTR region was generated and inserted into the psiCHECK-2 plasmid, name after KRAS-Mut-3’UTR (Fig. 4B). As shown in Fig. 4C, compared with transfected with mimics NC, co-transfection of KRAS-WT-3’UTR with hsa-miR-155 mimics led to a significant decrease in the luciferase activity. These regulatory effects were not observed at all with the KRAS-Mut-3’UTR.

For now, we have demonstrated that TGFβ1-induced noncanonical HH signaling repressed BMECs by promoting miR-155 expression. As mentioned, the MIR155HG was the precursor of miR-155, while HH signaling modulation gene expression was dependent on transcription factors Gli1 and Gli2 binding to promoters of target genes. Therefore, we aimed to identify the exact sites where Gli1/2 binds to the *mir155hg* promoter region. As shown in Fig. 4D, three potential Gli1/2 binding sites were predicted on the *mir155hg* promoter region (site 1–3). The *mir155hg* promoter region, including the full-length promoter region and a series of truncation and site-mutation, were cloned and constructed. Dual-luciferase reporter assays from both truncation and site mutations all indicated that the site 1, 5′-CAGGGAGGTCCA-3′ (from − 1088 to − 1077), was the Gli1/2 binding region on *mir155hg* promoter (Fig. 4E-G). These data confirm that rTGFβ1 increases miR-155 to suppress KRAS-ERK1/2 signaling, thereby repressing BMECs’ immune reaction.

### HH signaling agonist SAG repress BMECs immune reaction induced by meningitic *E. coli*

**Fig. 5:**
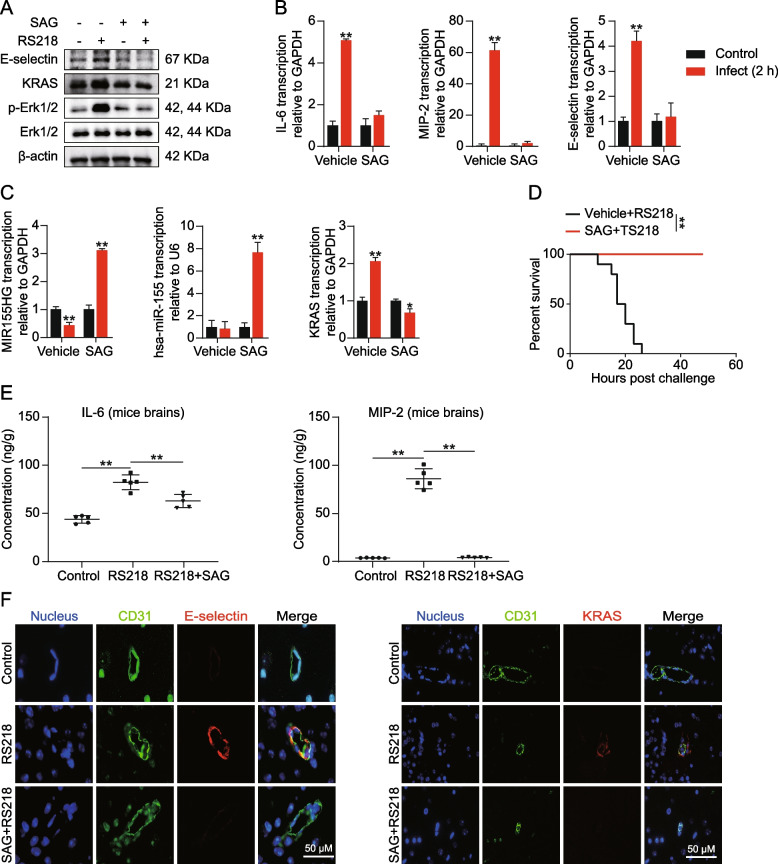
SAG repressed BMECs immune reaction and neuroinflammation of infected mice in vivo and in vitro. **A** Western blot detecting expression of E-selectin, KRAS and ERK1/2 phosphorylation in RS218 infected hBMECs with or without SAG pre-treatment (at 10 μM). **B** qPCR detecting expression alterations of IL-6, MIP-2, and E-selectin in RS218 infected hBMECs with or without SAG pre-treatment (at 10 μM). **p < 0.01. The qPCR assays were performed in triplicates, and results are presented as mean ± SEM. **C** qPCR detecting MIR155HG, miR-155, and KRAS expression upon RS218 infection in hBMECs. The cells were pretreated with or without SAG pre-treatment (at 10 μM). *p < 0.05, **p < 0.01. The qPCR assays were performed in triplicates, and results are presented as mean ± SEM. **D** Effects of the SAG pre-treatment at 10 mg/kg (for 12 h) on the survival of the mice after the challenge of RS218 (*n* = 10). **p < 0.01. **E** ELISA analysis of IL-6 and MIP-2 in brain lysates from RS218 challenged mice with or without SAG pre-treatment at 10 mg/kg. Data are presented as mean ± SEM from five individual mice in each group. **F** IF assays showing the E-selectin and KRAS expression in brains of mice challenged by RS218 with or without SAG pre-treatment (at 10 mg/kg). The E-selection and KRAS were stained in red. CD31 was specifically applied for labeling the micro-vessels in green. The cell nucleus was stained in blue with DAPI. Scale bar = 50 μm

Previously, we demonstrated that the TGF-HH axis repressed BMECs immune reaction induced through meningitic *E. coli* by modulating miR-155/KRAS/ERK signaling. Therefore, we speculated if activating HH signaling in BMECs with agonist SAG would also be effective. Here, we treated hBMECs with SAG before RS218 infection. As demonstrated in Fig. 5A-B, SAG suppressed ERK1/2 phosphorylation, IL-6, MIP-2, and E-selectin upregulation upon infection. Besides, the miR-155, and MIR155HG were upregulated in the presence of SAG, leading to the downregulation of downstream KRAS (Fig. 5C). These in vitro data suggested that activating HH signaling with SAG directly represses BMEC immune reaction by increasing miR-155 and suppressing KRAS/ERK signaling, similar to TGFβ1.

Subsequently, we validated SAG protective effects on mice infected with meningitic *E. coli* strain RS218. As presented in Fig. 5D, SAG treatment reduced mortality of mice challenged with RS218, and all 10 mice survived. Additionally, IL-6 and MIP-2 expression, and microglial activation in mice brains induced by *E. coli,* were significantly restrained by SAG (Figs. 1C and 5E). IF analysis indicated that E-selectin (marked with anti-E-selectin, red) expression in BMECs (marked with anti-CD31, green) was suppressed in mice injected with SAG. Furthermore, the KRAS (labeled with Cy3) expression was also reduced in BMECs (labeled with anti-CD31-FITC) of mice treated with SAG (Fig. 5F). The results indicated that HH agonist SAG has a protective effect on RS218-challenged mice. The reagent attenuated BMECs immune reaction and the subsequent neuroinflammation, thereby protecting infected animals from death.

## Discussion

BBB is a barrier separating CNS from peripheral circulation [27]. When infected with meningitic *E. coli*, the bacteria break down BMECs’ tight junctions and induce the release of inflammatory mediators, leading to barrier permeability rising and neuroinflammation [28–30]. Previously, we have demonstrated that astrocytes-derived TGFβ1, and exogenous TGFβ1, can upregulate tight junction protein ZO-1 and maintain BBB integrity, depending on a noncanonical HH signaling. Particularly, the exogenous rTGFβ1 rescued the mice infected with meningitic *E. coli* strain RS218, and HH signaling played the core role in this process [21]. In this study, we focused on the TGFβ1 immunity modulation in BBB. Through in vivo and in vitro experiments, we demonstrated that exogenous rTGFβ1 suppressed the immune reaction of BMECs and subsequently neuroinflammation upon infection. TGFβ1 induced HH signaling decreased ERK1/2 phosphorylation in BMECs resulting in reduced expression of the IL-6, MIP-2, and E-selectin expression. Further study revealed that HH signaling negatively regulated KRAS, upstream inducer of ERK1/2 signaling, through upregulating miR-155, thereby, decreasing ERK1/2 phosphorylation in BMECs. Additionally, we recognized the specific sites, on which miR-155 binding to *kras* gene 3’UTR regions and transcription factors Gli1/2 binding to *mir155hg* promoter. Combining these findings with those in our previous study, we concluded that exogenous TGFβ1 was capable of protecting animals from meningitic *E. coli* through both maintaining BBB integrity and repressing BMECs immune reaction.

In infectious diseases, BMECs responded actively to pathogens factors, releasing chemokines and cytokines to contribute to neuroinflammation. For example, during early infection of *P. falciparum*, the infected RBC-derived PFHRPII interacted with BMECs, activating endothelial NFκB signaling, leading to the release of inflammatory mediators such as IL-6 IL-1B CCL5 and IFNβ1 [31]. In *Streptococcus agalactiae* meningitis, bacterial Srr1 or Srr2 binds to fibrinogen on the surface of hBMECs, which largely contributes to *Streptococcus* CNS invasion and subsequent disease progression [32, 33]. Furthermore, reacted BMECs also mediated leukocytes to penetrate BBB into CNS, inducing an inflammatory storm. Specifically, selectins, carbohydrate-binding molecules that bind to fucosylated and sialylated glycoprotein ligands, induced the capture of leukocytes from the rapidly flowing blood, leading to leukocytes rolling on the apical endothelial cell surface [34]. Subsequently, with the assistance of ICAM1, VCAM1, and JAMs, the leukocytes crawl on the luminal surface of blood vessels and transmigrate through the endothelial barrier [35]. Considering the powerful regulatory role of BMECs in neuroinflammation, efforts have been made to combat the inflammatory storm by suppressing the endothelial immune reaction, and several herbal preservations targeting endothelial cells have been proved effective in reducing inflammation. For instance, Tanshinone IIA and Salvianolic acid A from the Chinese herb Salviae Miltiorrhizae Bunge inhibited adhesion molecules, chemokines, and eNOS expression in BMECs, expressing anti-inflammatory and immunoregulatory molecules during ischemic/reperfusion and multiple sclerosis treatment [36, 37]. Additionally, Tetramethylpyrazine isolated from Chinese Herb Medicine Ligustium wollichii Franchat was reportedly protected BBB against oxygen-glucose deprivation via repressing the Rho/ROCK signaling pathway in BMECs [38]. Currently, we demonstrated that TGFβ1 and SAG maintain BBB integrity and suppress BMECs immune reaction induced by meningitic *E. coli*, which indicated protective potential of HH signaling agonist onto CNS upon infection.

MicroRNAs (miRNAs) are endogenous ~ 22 nt RNAs targeting mRNAs for cleavage or translational repression, which played a regulatory role in the immunity process, particularly in BBB and CNS inflammatory reaction [39, 40]. For instance, miR-17-5p mediated leukemia inhibitory factor modulation of reactive astrocyte proliferation [41], and astrocytic miR-19b-3p participated in promoting the production of inflammatory cytokines in JEV infection [42]. In this work, we identified MIR155HG/miR-155 as the mediator of TGFβ1 repressing ERK1/2 signaling. MIR155HG, also known as the B-cell integration cluster, was transcribed from a gene located on chromosome 21q21 and consisted of three exons that span 1.5 kbp. There was an imperfectly base-paired stem-loop in exon 3 conserved across species, and it was a primary miRNA for miR-155 [43]. Previously, miR-155 was identified to participate in immune responses with complicated functions. For example, miR-155 in neutrophil microvesicles promoted inflammatory gene expression by targeting BCL6, an NFκB repressor, leading to vascular inflammation and atherogenesis [44]. In tumor-activated monocytes, induction of miR-155 suppressed C/EBPβ1 protein expression; cytokine production, and the tumor environments regulated the functional activities of monocytes by decreasing the miR-155 expression to release its translational inhibition of transcription factor C/EBPβ1 [45]. In CNS infection, miR-155 negatively regulated JEV-induced interferon regulatory factor 8 and complement factor H expression, which played a beneficial role in limiting virus replication in human microglial cells [46]. Here, we demonstrated miR-155 played as an anti-inflammatory factor in BMECs, and the potential as a target for cure neuroinflammation remained to be worked on.

For TGFβ1 effects relying on the inducted noncanonical HH signaling, we validated HH signaling agonist SAG immunosuppression effects on BMECs and the protective effects on mice challenged with RS218. Similar to rTGFβ1, SAG treatment suppressed the induction of ERK1/2 signaling and the downstream genes, IL-6, MIP-2, and E-selectin. Additionally, the mice treated with SAG survived RS218 attacking and avoided neuroinflammation. The data suggested SAG capability modulated CNS immunity function. As previously reported, HH signaling played a role in the peripheral immune system. For example, HH signaling in skin CD4+ T cells increased the expression of immunoregulatory genes and reduced the expression of inflammatory and chemokine genes via promoting FOXP3 expression, eventually mediated immune suppression [47]. Furthermore, HH signaling in macrophages was also promoted M2 polarization and mediated immunosuppression, allowing cancer tumor cells to escape from the immune system and apply immunotherapy [48]. In this work, the SAG protection of mice upon meningitic *E. coli* challenge might also associate with HH regulator role in peripheral immune cells, and the correlation is to be further explored.

## Conclusions

**Fig. 6:**
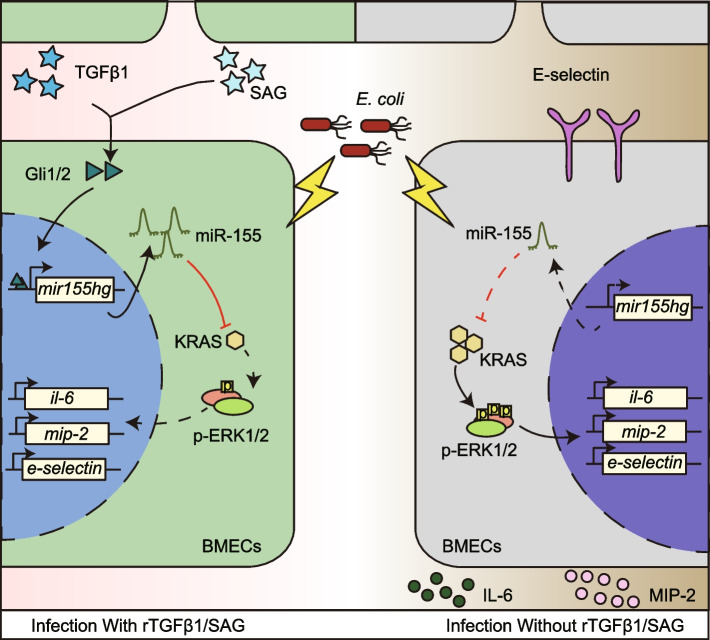
Schematic representation of the TGFβ1 or SAG immunosuppression effects on BMECs through upregulating miR-155 and negative regulating KRAS as well as downstream ERK1/2 signaling. The exogenous TGFβ1 or SAG triggered the HH signaling in *E. coli*-infected BMECs and upregulated intracellular miR-155. Further, the promoted miR-155 suppressed the ERK1/2 activation by negatively regulating KRAS, thus decreasing IL-6, MIP-2, and E-selectin expression

Our data indicated that the TGF-HH axis reversed the immune reaction in BMECs and neuroinflammation induced by meningitic *E. coli*. Here, we demonstrated TGFβ1 induced HH signaling downregulated KRAS via inducing miR-155; therefore, repressed ERK1/2 signaling activation in BMECs upon *E. coli* challenge (Fig. 6). Strikingly, we also demonstrated the HH signaling agonist SAG protection on mice with meningitic *E. coli* infection. This report illustrated the important therapeutic potential of HH signaling activation in curing infectious diseases. A better understanding of HH signaling effects and corresponding mechanisms are urgently needed for effective drug and therapy development in the era of increasing antibiotic resistance.

### Supplementary Information

**Additional file 1.**

**Additional file 2.**

## Data Availability

All data generated or analyzed during this study are included in this published article.

## Abbreviations

*E. coli*: *Escherichia coli*
CNS: Central nervous system
BBB: Blood-brain barrier
HH: Hedgehog
TGFβ1: Transforming growth factor beta 1
BMECs: Brain microvascular endothelial cells
rTGFβ1: Recombined TGFβ1
FBS: Fetal bovine serum
IF: Immunofluorescence
DAPI: 4′,6-diamidino-2-phenylindole
ELISA: Enzyme-linked immunosorbent assay
CDS: Coding sequence
ANOVA: Analysis of variance
MREs: miRNA response elements

## References

[1] McGill F, Heyderman RS, Panagiotou S, Tunkel AR, Solomon T (2016). Acute bacterial meningitis in adults. Lancet..

[2] Janowski A, Newland J. Of the phrensy: an update on the epidemiology and pathogenesis of bacterial meningitis in the pediatric population. F1000Res. 2017;6 F1000 Faculty Rev-86 10.12688/f1000research.8533.1 PMC5288681 28184287

[3] Kim KS. Human meningitis-associated *Escherichia coli*. EcoSal Plus. 2016;7 10.1128/ecosalplus.ESP-0015-2015. 10.1128/ecosalplus.esp-0015-2015 PMC4881430 27223820

[4] Kim KS (2003). Pathogenesis of bacterial meningitis: from bacteraemia to neuronal injury. Nat Rev Neurosci..

[5] Yang R, Wang J, Wang F, Zhang H, Tan C, Chen H, Wang X (2023). Blood-brain barrier integrity damage in bacterial meningitis: the underlying link, mechanisms, and therapeutic targets. Int J Mol Sci..

[6] Keaney J, Campbell M (2015). The dynamic blood-brain barrier. FEBS J..

[7] Ludewig P, Winneberger J, Magnus T (2019). The cerebral endothelial cell as a key regulator of inflammatory processes in sterile inflammation. J Neuroimmunol..

[8] Yang RC, Huang K, Zhang HP, Li L, Zhang YF, Tan C, Chen HC, Jin ML, Wang XR (2022). SARS-CoV-2 productively infects human brain microvascular endothelial cells. J Neuroinflammation..

[9] Erickson MA, Banks WA (2018). Neuroimmune axes of the blood-brain barriers and blood-brain interfaces: bases for physiological regulation, disease states, and pharmacological interventions. Pharmacol Rev..

[10] Pachter JS, de Vries HE, Fabry Z (2003). The blood-brain barrier and its role in immune privilege in the central nervous system. J Neuropathol Exp Neurol..

[11] Gimenez MA, Sim J, Archambault AS, Klein RS, Russell JH (2006). A tumor necrosis factor receptor 1-dependent conversation between central nervous system-specific T cells and the central nervous system is required for inflammatory infiltration of the spinal cord. Am J Pathol..

[12] Bell MD, Taub DD, Kunkel SJ, Strieter RM, Foley R, Gauldie J, Perry VH (1996). Recombinant human adenovirus with rat MIP-2 gene insertion causes prolonged PMN recruitment to the murine brain. Eur J Neurosci..

[13] McCarthy RC, Kosman DJ (2014). Activation of C6 glioblastoma cell ceruloplasmin expression by neighboring human brain endothelia-derived interleukins in an *in vitro* blood-brain barrier model system. Cell Commun Signal..

[14] Stankiewicz JM, Brass SD (2009). Role of iron in neurotoxicity: a cause for concern in the elderly?. Curr Opin Clin Nutr Metab Care..

[15] Ma XJ, Cheng JW, Zhang J, Liu AJ, Liu W, Guo W, Shen FM, Lu GC (2012). E-selectin deficiency attenuates brain ischemia in mice. CNS Neurosci Ther..

[16] Chen Y, Ruetzler C, Pandipati S, Spatz M, McCarron RM, Becker K, Hallenbeck JM (2003). Mucosal tolerance to E-selectin provides cell-mediated protection against ischemic brain injury. Proc Natl Acad Sci U S A..

[17] Li AG, Wang D, Feng XH, Wang XJ (2004). Latent TGFbeta1 overexpression in keratinocytes results in a severe psoriasis-like skin disorder. EMBO J..

[18] Han G, Li F, Singh TP, Wolf P, Wang XJ (2012). The pro-inflammatory role of TGFβ1: a paradox?. Int J Biol Sci..

[19] Ungefroren H, Gieseler F, Kaufmann R, Settmacher U, Lehnert H, Rauch BH (2018). Signaling crosstalk of TGF-β/ALK5 and PAR2/PAR1: a complex regulatory network controlling fibrosis and cancer. Int J Mol Sci..

[20] Butz H, Racz K, Hunyady L, Patocs A (2012). Crosstalk between TGF-β signaling and the microRNA machinery. Trends Pharmacol Sci..

[21] Fu JY, Li L, Huo D, Yang RC, Yang B, Xu BJ, Yang XP, Dai MH, Tan C, Chen HC, Wang XR (2021). Meningitic α-hemolysin aggravates blood-brain barrier disruption targeting TGFβ1-triggered hedgehog signaling. Mol Brain..

[22] Fu JY, Li L, Huo D, Zhi SL, Yang RC, Yang B, Xu BJ, Zhang T, Dai MH, Tan C (2021). Astrocyte-derived TGFβ1 facilitates blood-brain barrier function via non-canonical hedgehog signaling in nrain microvascular endothelial cells. Brain Sci..

[23] Wang X, Maruvada R, Morris AJ, Liu JO, Wolfgang MJ, Baek DJ, Bittman R, Kim KS (2016). Sphingosine 1-phosphate activation of EGFR as a novel target for meningitic *Escherichia coli* penetration of the blood-brain barrier. PLoS Pathog..

[24] Hoffmann I, Eugene E, Nassif X, Couraud PO, Bourdoulous S (2001). Activation of ErbB2 receptor tyrosine kinase supports invasion of endothelial cells by *Neisseria meningitidis*. J Cell Biol..

[25] Yuan J, Dong X, Yap J, Hu J (2020). The MAPK and AMPK signalings: interplay and implication in targeted cancer therapy. J Hematol Oncol..

[26] Chakraborty C, Sharma AR, Patra BC, Bhattacharya M, Sharma G, Lee SS (2016). MicroRNAs mediated regulation of MAPK signaling pathways in chronic myeloid leukemia. Oncotarget..

[27] Pardridge WM (2005). Molecular biology of the blood-brain barrier. Mol Biotechnol..

[28] Yang R, Liu W, Miao L, Yang X, Fu J, Dou B, Cai A, Zong X, Tan C, Chen H, Wang X (2016). Induction of VEGFA and Snail-1 by meningitic *Escherichia coli* mediates disruption of the blood-brain barrier. Oncotarget..

[29] Yang RC, Qu XY, Xiao SY, Li L, Xu BJ, Fu JY, Lv YJ, Amjad N, Tan C, Kim KS (2019). Meningitic *Escherichia coli*-induced upregulation of PDGF-B and ICAM-1 aggravates blood-brain barrier disruption and neuroinflammatory response. J Neuroinflammation..

[30] Liu WT, Lv YJ, Yang RC, Fu JY, Liu L, Wang H, Cao Q, Tan C, Chen HC, Wang XR (2018). New insights into meningitic *Escherichia coli* infection of brain microvascular endothelial cells from quantitative proteomics analysis. J Neuroinflammation..

[31] Pais TF, Penha-Goncalves C (2018). Brain endothelium: the "innate immunity response hypothesis" in cerebral malaria pathogenesis. Front Immunol..

[32] Seo HS, Mu R, Kim BJ, Doran KS, Sullam PM (2012). Binding of glycoprotein Srr1 of *Streptococcus agalactiae* to fibrinogen promotes attachment to brain endothelium and the development of meningitis. PLoS Pathog..

[33] Seo HS, Minasov G, Seepersaud R, Doran KS, Dubrovska I, Shuvalova L, Anderson WF, Iverson TM, Sullam PM (2013). Characterization of fibrinogen binding by glycoproteins Srr1 and Srr2 of *Streptococcus agalactiae*. J Biol Chem..

[34] Ley K (2003). The role of selectins in inflammation and disease. Trends Mol Med..

[35] Vestweber D (2015). How leukocytes cross the vascular endothelium. Nat Rev Immunol..

[36] Yang X, Yan J, Feng J (2016). Treatment with tanshinone IIA suppresses disruption of the blood-brain barrier and reduces expression of adhesion molecules and chemokines in experimental autoimmune encephalomyelitis. Eur J Pharmacol..

[37] Mahmood Q, Wang GF, Wu G, Wang H, Zhou CX, Yang HY, Liu ZR, Han F, Zhao K (2017). Salvianolic acid a inhibits calpain activation and eNOS uncoupling during focal cerebral ischemia in mice. Phytomedicine..

[38] Yang G, Qian C, Wang N, Lin C, Wang Y, Wang G, Piao X (2017). Tetramethylpyrazine protects against oxygen-glucose deprivation-induced brain microvascular endothelial cells injury via rho/rho-kinase signaling pathway. Cell Mol Neurobiol..

[39] Bartel DP (2004). MicroRNAs: genomics, biogenesis, mechanism, and function. Cell..

[40] Yang R, Yang B, Liu W, Tan C, Chen H, Wang X (2023). Emerging role of non-coding RNAs in neuroinflammation mediated by microglia and astrocytes. J Neuroinflammation..

[41] Hong P, Jiang M, Li H (2014). Functional requirement of dicer1 and miR-17-5p in reactive astrocyte proliferation after spinal cord injury in the mouse. Glia..

[42] Ashraf U, Zhu B, Ye J, Wan S, Nie Y, Chen Z, Cui M, Wang C, Duan X, Zhang H (2016). MicroRNA-19b-3p modulates Japanese encephalitis virus-mediated inflammation via targeting RNF11. J Virol..

[43] Elton TS, Selemon H, Elton SM, Parinandi NL (2013). Regulation of the MIR155 host gene in physiological and pathological processes. Gene..

[44] Gomez I, Ward B, Souilhol C, Recarti C, Ariaans M, Johnston J, Burnett A, Mahmoud M, Luong LA, West L (2020). Neutrophil microvesicles drive atherosclerosis by delivering miR-155 to atheroprone endothelium. Nat Commun..

[45] He M, Xu Z, Ding T, Kuang DM, Zheng L (2009). MicroRNA-155 regulates inflammatory cytokine production in tumor-associated macrophages via targeting C/EBPbeta. Cell Mol Immunol..

[46] Pareek S, Roy S, Kumari B, Jain P, Banerjee A, Vrati S (2014). MiR-155 induction in microglial cells suppresses Japanese encephalitis virus replication and negatively modulates innate immune responses. J Neuroinflammation..

[47] Papaioannou E, Yanez DC, Ross S, Lau CI, Solanki A, Chawda MM, Virasami A, Ranz I, Ono M, O'Shaughnessy RFL, Crompton T (2019). Sonic hedgehog signaling limits atopic dermatitis via Gli2-driven immune regulation. J Clin Invest..

[48] Petty AJ, Li A, Wang X, Dai R, Heyman B, Hsu D, Huang X, Yang Y (2019). Hedgehog signaling promotes tumor-associated macrophage polarization to suppress intratumoral CD8^+^ T cell recruitment. J Clin Invest..

