# Supplementary material for: TGFβ1-induced hedgehog signaling suppresses the immune response of brain microvascular endothelial cells elicited by meningitic Escherichia coli
Source: Cell Commun Signal. 2024 Feb 15;22:123. doi: 10.1186/s12964-023-01383-y (PMC10868028; doi:10.1186/s12964-023-01383-y)
Supplement: Supplementary file 2 — Additional file 1. [file 12964_2023_1383_MOESM1_ESM.docx]

**Table S1. Primers used for the qPCR assays**

| **Primer name** | **Sequence (5’ to 3’)** |
| --- | --- |
| *il-6*-F | CCTTCGGTCCAGTTGCCTTCT |
| *il-6*-R | GAGGTGAGTGGCTGTCTGTGT |
| *mip-2*-F | GCTTGTCTCAACCCCGCATC |
| *mip-2*-R | TGGATTTGCCATTTTTCAGCATCTT |
| *e-selectin*-F | CAGCAAAGGTACACACACCTG |
| *e-selectin*-R | CAGACCCACACATTGTTGACTT |
| *mir155hg*-F | GAGTGCTGAAGGCTTGCTGT |
| *mir155hg*-R | TTGAACATCCCAGTGACCAG |
| *kras*-F | ACAGAGAGTGGAGGATGCTTT |
| *kras*-R | TTTCACACAGCCAGGAGTCTT |
| *gapdh*-F | CAACAGCCTCAAGATCATCAG |
| *gapdh*-R | GAGTCCTTCCACGATACCA |
| *u6*-F | CTCGCTTCGGCAGCACA |
| *u6*-R | AACGCTTCACGAATTTGCGT |
