# Supplementary material for: TGFβ1-induced hedgehog signaling suppresses the immune response of brain microvascular endothelial cells elicited by meningitic Escherichia coli
Source: Cell Commun Signal. 2024 Feb 15;22:123. doi: 10.1186/s12964-023-01383-y (PMC10868028; doi:10.1186/s12964-023-01383-y)
Supplement: Supplementary file 3 — Additional file 2. [file 12964_2023_1383_MOESM2_ESM.docx]

**Table S2.** Primers for CDSs cloning and Promotor region amplification in the dual-luciferase reporter assays

| **Primer name** | **Sequence (5’ to 3’)** |
| --- | --- |
| Gli1-CDS -F | CCCAAGCTTATGTTCAACTCGATGACCCCAC |
| Gli1-CDS -R | GATATCTTAGGCACTAGAGTTGAGGAATTCTG |
| Gli2-CDS -F | CCCAAGCTTATGGAGACGTCTGCCTCAGCC |
| Gli2-CDS -R | CCGGAATTCCTAGGTCATCATGTTCAGGAACTTG |
| *mir155hg*-promo-F | CCCCTCGAGTCTCTGTGCTCAGTTTCCTCATC |
| *mir155hg*-promo-R | CCGAAGCTTGCCTTGCCTCGCCAGTTAA |
| *mir155hg*-promo truncation1-F | CCCCTCGAGATGAGTTACTCGACTAGGCTTGTAG |
| *mir155hg*-promo truncation1-R | CCGAAGCTTGCCTTGCCTCGCCAGTTAA |
| *mir155hg*-promo truncation2-F | CCCCTCGAGCCGTCATTTGAAGGCGTTTCC |
| *mir155hg*-promo truncation2-R | CCGAAGCTTGCCTTGCCTCGCCAGTTAA |
| *mir155hg*-promo mutation1-F | CCTTGACTACAAATGTGAAGTACTGGATACTTGCAATGCAATCC |
| *mir155hg*-promo mutation1-R | TCACATTTGTAGTCAAGGGCACAAGACATTTTAGAA |
| *mir155hg*-promo mutation2-F | GAACTACAAATGTGATCCTCTCGGGCTCCCTGC |
| *mir155hg*-promo mutation2-R | GGATCACATTTGTAGTTCCTCCTGGACCGAATCAGC |
| *mir155hg*-promo mutation3-F | CTCCTACAAATGTGAAAGAAGTCGCCACTTCCCCC |
| *mir155hg*-promo mutation3-R | CTTTCACATTTGTAGGAGTGTCTCTACTCTCTCCTTGCAGG |
